# The role of the environment in transmission of *Dichelobacter nodosus* between ewes and their lambs

**DOI:** 10.1016/j.vetmic.2015.04.010

**Published:** 2015-08-31

**Authors:** Mohd Muzafar, Leo A. Calvo-Bado, Laura E. Green, Edward M. Smith, Claire L. Russell, Rose Grogono-Thomas, Elizabeth M.H. Wellington

**Affiliations:** a School of Life Sciences, University of Warwick, Gibbet Hill Road, Coventry CV4 7AL, UK; b Department of Clinical Veterinary Sciences, University of Bristol, Langford House, Langford BS40 5DU, UK

**Keywords:** *D. nodosus*, Footrot, Pathogen transmission, Disease reservoirs

## Abstract

*Dichelobacter nodosus* (*D. nodosus*) is the essential causative agent of footrot in sheep. The current study investigated when *D. nodosus* was detectable on newborn lambs and possible routes of transmission. Specific qPCR was used to detect and quantify the load of *D. nodosus* in foot swabs of lambs at birth and 5–13 h post-partum, and their mothers 5–13 h post-partum; and in samples of bedding, pasture, soil and faeces. *D. nodosus* was not detected on the feet of newborn lambs swabbed at birth, but was detected 5–13 h after birth, once they had stood on bedding containing naturally occurring *D. nodosus*. Multiple genotypes identified by cloning and sequencing a marker gene, *pgrA*, and by multi locus variable number tandem repeat analysis (MLVA) of community DNA from swabs on individual feet indicated a mixed population of *D. nodosus* was present on the feet of both ewes and lambs. There was high variation in *pgrA* tandem repeat number (between 3 and 21 repeats), and multiple MLVA types. The overall similarity index between the populations on ewes and lambs was 0.45, indicating moderate overlap. Mother offspring pairs shared some alleles but not all, suggesting lambs were infected from sources(s) other than just their mother's feet. We hypothesise that *D. nodosus* is transferred to the feet of lambs via bedding containing naturally occurring populations of *D. nodosus*, probably as a result of transfer from the feet of the group of housed ewes. The results support the hypothesis that the environment plays a key role in the transmission of *D. nodosus* between ewes and lambs.

## Highlights

• Lambs’ feet were *D. nodosus*-negative at birth.
• However *D. nodosus* was detected on lambs’ feet within 5–13 h of birth.
• Multiple *pgrA* and MLVA alleles were detected on the feet of ewes and lambs.
• *D. nodosus* on lambs’ feet originated from sources other than just their mother's feet.
• The environment plays a key role in *D. nodosus* transmission between ewes and lambs.

## 1 Introduction

Footrot is an economically important disease of sheep. The aerotolerant anaerobe *Dichelobacter nodosus* (*D. nodosus*) is the essential causative agent (Beveridge, 1941) and *Fusobacterium necrophorum* has been suggested as a secondary bacterium after the development of disease (Beveridge, 1941; Witcomb et al., 2014). The disease is present worldwide and accounts for annual losses of between £24 and £84 million to the UK sheep industry alone (Nieuwhof and Bishop, 2005; Wassink et al., 2010). The severity of ovine footrot can vary from mild interdigital dermatitis (synonymous with benign footrot in Australian research) to virulent footrot causing severe under-running of the hoof horn with separation from the underlying tissue (Stewart, 1989). *D. nodosus* can be detected on the feet of sheep with no sign of disease (Calvo-Bado et al., 2011b; Witcomb et al., 2014) but the load is higher both before and during episodes of interdigital dermatitis and virulent footrot than on healthy feet (Witcomb et al., 2014).

Temporal clustering of footrot between mothers and offspring was observed in a state transition study of factors associated with development of, and recovery from, footrot. Given that families cluster spatially this suggests spatiotemporal transmission of *D. nodosus* between family members (Kaler et al., 2010). *D. nodosus* has been isolated from pasture and barns where sheep are kept, indicating that contamination of the environment occurs (Witcomb, 2012). Contaminated holding areas have also been shown to cause disease in sheep put into such environments up to 2 weeks from initial seeding (Beveridge, 1941; Whittington, 1995). Recent work has indicated that *D. nodosus* can survive up to 14 days at 5 °C in soil, and at least 24 days when hoof material was present (Cederlof et al., 2013) and under certain conditions, *D. nodosus* has survived for at least 40 days in soil microcosms (unpublished data), however, further work is required to determine if survival is at a dose that could cause disease in sheep.

Multiple strains of *D. nodosus* detected by serogroup typing have been reported to co-exist in individual feet during subclinical and clinical infections (Claxton et al., 1983; Hindmarsh and Fraser, 1985; Jelinek et al., 2000; Moore et al., 2005). Molecular detection of strain differences is now possible using typing the *pgr* locus and by MLVA of *D. nodosus* (Calvo-Bado et al., 2011a; Russell et al., 2014).

The aims of this study were to investigate whether *D. nodosus* was present on the feet of newborn lambs at or after birth and the potential role played by the environment in pathogen transmission.

## 2 Materials and methods

### 2.1 Selection of animals

In April 2011 10 ewes with no clinical signs of disease and one lamb per ewe were convenience selected from a flock of 99 Mule and Suffolk crossbred ewes. Ewes were housed on the 28th March 2011, and samples collected on the 1st–6th April 2011 (Supplementary Table 1). Lambs were born in a large communal straw bedded pen, ewes and their lambs were moved to individual pens once the ewe had given birth to all her lambs. Sampled lambs were marked with tape so they could be identified for subsequent sampling.

### 2.2 Collection of environmental and foot swab samples

Environmental samples were taken in March prior to lambing and included swab samples of 30 fresh hoof prints in soil, four soil samples from the area around water containers, 10 samples of faecal material on the ground and compacted in the interdigital space and three straw samples collected from the storage area. In April, 10 straw bedding samples were collected from the communal pen where pregnant ewes were housed. All samples were stored at 4 °C for transportation and at −80 °C until analysed. All four feet of each lamb was swabbed using sterile cotton swabs (EUROTUBO collection swab; Delta lab, Rubi, Spain) directly after birth and before the lamb touched the ground. The lamb and its dam were sampled 5–13 h later once the lamb had stood and been transferred, with its mother, to an individual pen. Swabs were stored at 4 °C for transportation and at −80 °C on arrival at the laboratory.

### 2.3 Detection limit assay by direct PCR and nested PCR from swabs

The *D. nodosus* strain VCS1703A was used as a positive control for all PCR reactions. To determine the PCR detection limits, cells were harvested from a 5 d culture grown on 2% hoof agar, and 10-fold serial dilutions (10^−1^ to 10^−10^) were made in triplicate in sterile phosphate buffered saline (PBS). The numbers of cells in the initial concentration and all dilutions were counted using a haemocytometer. Sterile swabs were inoculated with 500 μl of each dilution, and frozen at −20 °C to produce swabs containing a known bacterial load. Microbial DNA was extracted from swabs as described below and the DNA used to determine assay detection limits.

### 2.4 DNA extraction from swabs

Total genomic DNA was extracted using the NucleoSpin Tissue Kit (Macherey-Nagel, GmbH and Co, Düren, Germany) with modifications. Swabs were thawed at 4 °C and 400 μl of buffer T1 was added followed by 40 μl of proteinase K. The samples were vortexed twice for 5 s and incubated for 10 min at 56 °C. The mixtures were transferred to microcentrifuge tubes and 400 μl of buffer B3 was added. The samples were vortexed twice for 5 s and incubated for 5 min at 70 °C then allowed to cool before adding 400 μl of 100% ethanol. The samples were again vortexed twice and the supernatant transferred to a NucleoSpin Tissue column and centrifuged at 11,000 × *g* for 1 min. The flow-through was discarded, the membrane was washed with 500 μl of buffer B5 and centrifuged at 11,000 × *g* for 1 min. The flow-through was again discarded, the column was washed with 600 μl of buffer B5 and centrifuged at 11,000 × *g* for 1 min. The flow-through was again discarded and the membrane dried by centrifugation at 11,000 × *g* for 1 min to remove residual ethanol. The DNA was eluted into 40 μl of elution buffer, warmed to 70 °C and centrifuged at 11,000 × *g* for 1 min and the resultant DNA was stored at −20 °C.

### 2.5 DNA extraction from soil and faeces

DNA was extracted from soil and faecal samples (one gram each) using the Fast DNA Spin Kit for soil (QBiogene, Carlsbad, CA, USA) according to the manufacturer's instructions, and eluted in 70 μl DES (DNase/Pyrogen Free Water). Sterile soil (autoclaved twice at 121 °C for 15 min) was used as negative controls for each set of extractions. The resultant DNA was stored at −20 °C.

### 2.6 DNA extraction from bedding

One gram of each of 10 bedding samples was thawed, and suspended in 40 ml of transport buffer (sterile PBS containing 20 mM Na_2_ EDTA; pH 8.0). The samples were shaken for 1 h at 37 °C followed by centrifugation for 15 min at 13,523 × *g* at 4 °C. The supernatant was removed and the pellet resuspended in 2 ml sterile PBS. DNA was extracted from 200 μl of this solution using the NucleoSpin Blood kit (Macherey-Nagel, GmbH and Co, Düren, Germany) according to the manufacturer's recommendations, and DNA was stored at −20 °C.

### 2.7 End point and nested PCR

PCR amplifications were performed using an Eppendorf vapo.protect Mastercycler (Eppendorf, Hamburg, Germany). Each 50 μl reaction contained 25 μl Promega PCR master mix (Promega, Southampton, UK), 1.0 μl each of primers Cc and Ac [10 mM] (La Fontaine et al., 1993) (Table 1), 2.5 μl of dimethyl sulfoxide (Fisher Scientific, Leicestershire, UK), 2 μl bovine serum albumin [10 mg ml^−1^] (Sigma–Aldrich Ltd., Poole, Dorset, UK), 16.5 μl of nuclease free water and 2 μl of template DNA. For direct detection of *D. nodosus*, PCR was performed using Cc and Ac primers (La Fontaine et al., 1993) (Table 1) under the following conditions: 1 cycle of 95 °C for 2 min, 40 cycles of 95 °C for 1 min, 60 °C for 45 s and 72 °C for 2 min and a final extension step of 72 °C for 5 min. Samples that were negative using this approach, were tested further using nested PCR. In the first round *16S rRNA* universal primers 27F and 1525R (Table 1) (Baker et al., 2003; Lane, 1991) were used in the conditions described above but with an annealing temperature of 55 °C, 1 μl of this product was used in the second round of PCR as described above. The PCR products were visualised under UV light.

### 2.8 Quantitative PCR of *D. nodosus*

The load of *D. nodosus* was determined using the Applied Biosystems 7500 Fast real-time detection system (Applied Biosystems, Warrington, UK). The qPCR targeted the *rpoD* gene (RNA polymerase sigma 70 factor, single copy number in *D. nodosus* genome) as described previously (Calvo-Bado et al., 2011b). All PCR reactions were performed in triplicate and each contained 12.5 μl TaqMan Universal Master Mix (Applied Biosystems, Warrington, UK), 2.25 μl each of *rpoDF* and *rpoDR* [10 pmol μl^−1^], 0.625 ml *rpoD* probe [10 pmol μl^−1^] (Table 1), 1.25 μl bovine serum albumin [10 mg ml^−1^], 5.375 μl nuclease free water and 1 μl of template DNA. DNA dilutions of 1:10 were also used to investigate potential inhibitors of the reaction. In addition, known concentrations of target DNA were added to negative samples as internal controls. A non-template control (nuclease free water) was included in triplicate in all PCR reactions. The reaction was carried out under the following conditions: one cycle at 50 °C for 2 min, one cycle at 95 °C for 10 min, followed by 40 cycles at 95 °C for 15 s and 55 °C for 1 min. The *rpoD* copy number was estimated based on the standard curve obtained from analysis of 10-fold serial dilutions of DNA extracted from *D. nodosus* strain VCS1703A.

### 2.9 Quantitative PCR assay for *pgrA* and *pgrB*

A fluorescent PCR was designed to increase the sensitivity of detection of *pgrA* and *B* (Table 1). The specificity of the *pgrA* and *B* primers was tested against genomic DNA isolated from 11 bacterial species and from a range of diverse environmental samples. *D. nodosus* DNA from VCS1703A was used as a positive control and genomic DNA from *Escherichia coli* and sterile water as negative and no target controls respectively (Supplementary Table 2). The cycling conditions were modified slightly from those described above by reducing the number of cycles to 38 and 36 for *pgrA* and *pgrB* respectively.

### 2.10 Cloning and sequencing of *pgrA* amplicons

The *pgrA* gene was amplified using the primers *pgrA*F1 and *pgrA*R1 (Table 1; (Calvo-Bado et al., 2011a)) from the DNA extracted from the foot swabs of five ewes (*n* = 14 feet) and their lambs (*n* = 10 feet) and cloned using the TOPO TA Cloning Kit (Invitrogen Ltd., Paisley, UK). The cloning reactions were set up following the manufacturer's recommendations. Transformations were carried out using chemically competent (TOP10) *E. coli* cells (Invitrogen Ltd., Paisley, UK) with 50 μg ml^−1^ kanamycin. One-hundred microliters of the resulting solution was cultured on LB plates containing 50 μg ml^−1^ kanamycin. Fifty colonies per sample were inoculated into individual wells of a 96-well plate, each containing 50 μl sterile water. The samples were heated to 75 °C for 10 min, and 1 μl of this solution was used as a template for PCR, resulting in analysis of 1200 transformants. The PCR products were run on a 1% high resolution agarose gel and visualised under UV light. The clones that showed variation in size within each foot were inoculated into LB media to provide sufficient biomass for plasmid DNA extraction using the Qiagen MiniPrep kit (Qiagen, West Sussex, UK). The plasmid DNA was digested using EcoR1 and sequenced by GATC Biotech (London, UK) using the supplied M13f and M13r primers (Invitrogen, Paisley, UK; Table 1). All sequences were deposited in GenBank database under accession numbers KR105403–KR105413.

### 2.11 Multi Locus VNTR analysis

The four *D. nodosus* tandem repeat (DNTR) loci were amplified individually as described previously (Russell et al., 2014) (Table 1) from the DNA isolated from the feet of all ewes and lambs. Primer specificity was tested against genomic DNA isolated from 11 bacterial species and from a range of diverse environmental samples (Supplementary Table 2). *D. nodosus* strain VCS1703A was included as a positive control for all PCR reactions. The amplified products from the community DNA were pooled in the ratio of 1:1:1:1 and submitted for fragment analysis to the University of Dundee (DNA Sequencing & Services, Dundee, Scotland). GeneScan 1200 LIZ dye (Applied Biosystems, Warrington, UK) was used as a size standard in fragment analysis. The data obtained were processed using Peak Scanner Software (Applied Biosystems, Warrington, UK) and analysed using T-REX (Culman et al., 2009) with a minimum fragment length cut off values of 300 bp and 500 bp for DNTR10 and DNTR19 respectively, peak height baseline threshold of 40 and bin range of 4 bp.

### 2.12 Statistical analysis

To determine the strain overlap between all ewes and lambs defined by MLVA, the coincidence index of overlap was calculated using the formula: *C* = 2*B*/(*E* + *L*), where *C* = coincidence index of overlap, *B* = occurrence of the same allele in ewes and lambs, *E* = occurrence of the allele in ewes, *L* = occurrence of the allele in lambs. Results can range from *C* = 0, no overlap between ewes and lambs to *C* = 1, identical strains occur in both ewes and lambs (Dice, 1945).

The *rpoD* copy number in ewes and lambs was not normally distributed. Therefore, a Mann–Whitney U test (Mann and Whitney, 1947) was used to test for differences in copy number between ewes and lambs.

## 3 Results

### 3.1 *D. nodosus* copy number detection limit and its persistence in the environment

**Fig. 1:**
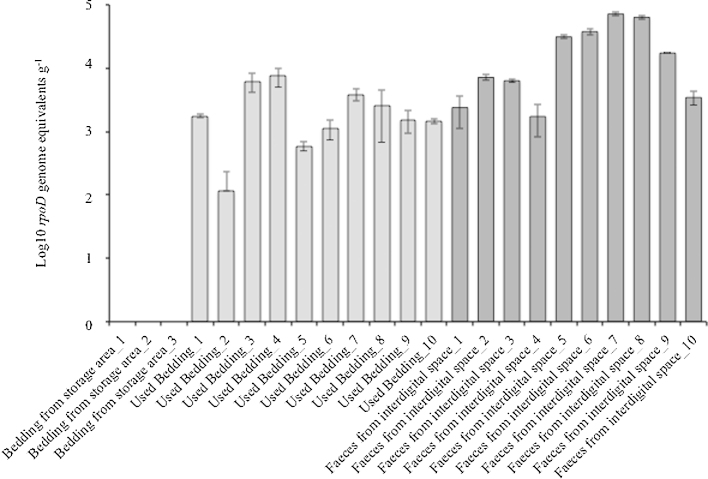
Presence of *D. nodosus* in the environmental samples. Absolute quantification of *rpoD* gene in bedding samples from the storage area, used bedding samples and faeces compacted within the interdigital space. Each bar is the average of triplicate analyses, error bars represent ± standard deviation.

End point PCR and qPCR data of the inoculated swabs suggested that the minimum detection level was 10^4^ cells and 10^2^ *rpoD* genome equivalents (i.e. 10^2^ cells) per swab respectively assuming 100% DNA recovery. Below this concentration, detection was not reproducible. *D. nodosus* was detected by end point PCR in 5/10 faecal samples/balls from the interdigital space, 2/10 straw bedding samples collected after ewes were housed, 5/30 fresh hoof prints and 2/4 soil samples taken from the areas surrounding water containers. However qPCR analysis revealed that *D. nodosus* was present at loads of 10^2^–10^4^ *rpoD* genome equivalents per gram in all the used straw bedding samples and 10^3^–10^4^ *rpoD* genome equivalents per gram in all the faecal samples. Quantitative PCR also confirmed that *D. nodosus* was not detectable in the three stored straw samples (Fig. 1).

### 3.2 Quantification of *D. nodosus* in ewes and lambs

**Fig. 2:**
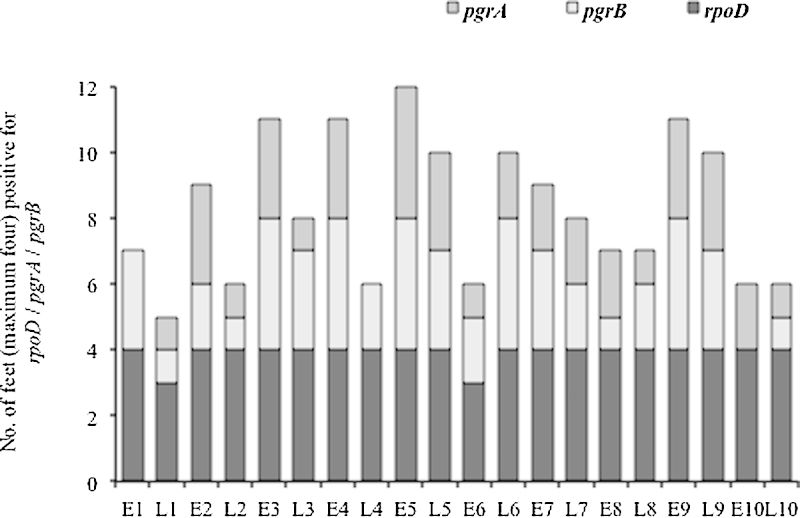
Detection of *pgr* variants in the community DNA. Presence of *pgrA*, *pgrB* and *rpoD* on the feet of 10 ewes and 10 lambs. *pgrA*/*B* was absent/below the detection limit in the samples where no data is shown. (E = Ewe; L = Lamb).

*D. nodosus* was not detected in lambs’ feet at birth but was detected in all lambs and ewes 5–13 h later after their feet had touched the floor initially in a large communal pen, and subsequently in an individual pen. *D. nodosus* was detected on 39/40 (97.5%) of ewes’ feet and 39/40 (97.5%) of lambs’ feet (Fig. 2). Whilst overall the population loads were significantly higher in ewes than lambs (Mann–Whitney U test; *p*-value < 0.001); analysis of ewe/lamb pairs suggested only ewes 2, 3 and 5 had a higher load than their lambs. The *D. nodosus* load ranged from 10^3^ to 10^5^ *rpoD* genome equivalents per swab in lambs and 10^2^–10^7^ *rpoD* genome equivalents per swab in ewes (Supplementary Fig. 1).

### 3.3 Detection of *pgr* variants in the community DNA

*pgrA* was detected on 23/40 (57.5%) of ewes’ feet and 15/40 (37.5%) of lambs’ feet, whereas *pgrB* was detected on 27/40 (67.5%) of ewes’ feet and 22/40 (55%) of lambs’ feet (Fig. 2). Both variants *pgrA* and *pgrB* were detected on eight ewes and nine lambs.

Forty-two *pgrA* clones were sequenced from 14 foot swabs from five ewes and 10 foot swabs from five lambs. This resulted in the detection of 11 variants containing 3–21 tandem repeats in the R1 region, with 2–6 variants per animal (Table 2). Multiple *pgrA* variants with varying numbers of tandem repeats were observed in a single foot swab (data not shown).

### 3.4 Molecular typing MLVA from the community DNA

Alleles at *D. nodosus* tandem repeat DNTR19 were detected on the feet of six ewes and their lambs whereas for DNTR10 alleles were only detected on two ewe/lamb pairs (Table 3) (Supplementary Fig. 2). The fluorescent data for DNTR09 was below the peak height threshold level (40 fluorescence units) and DNTR02 primers demonstrated some non-specific binding, so were excluded from the analysis. For both loci (DNTR10 and DNTR19), one or two alleles occurred in lambs, but there was greater diversity in ewes, with up to six alleles detected (Table 3). As detectable diversity in ewes increased, so did the likelihood of detecting the same strain on its offspring. The overall coincidence index of overlap between ewes and lambs was 0.45.

## 4 Discussion

In the current study we demonstrated, for the first time, that lambs are born free from *D. nodosus* (given the sensitivity of the tests used) but that we were able to detect high levels of *D. nodosus* on foot swabs within a few hours of birth. The most likely route of transmission is from standing on contaminated bedding in communal pens, however, recent work (Witcomb, 2012) indicated that *D. nodosus* can be detected in the mouths of ewes, so transmission might also have occurred as the lamb was being cleaned by the ewe after birth. Quantitative PCR with specific primers is widely used to determine bacterial load from swabs (Fredricks et al., 2009; Jaton et al., 2006; Lund et al., 2004) especially when studying disease development over time (Srinivasan et al., 2010). Here, as elsewhere (Witcomb et al., 2014) qPCR of *rpoD* was used to determine *D. nodosus* load on the feet of ewes and lambs. It was striking that such a large *D. nodosus* population was present on the feet of lambs within a few hours of birth. This suggests that *D. nodosus* might be an early coloniser of lambs’ feet, although, it could be that naïve feet are simply colonized by the first bacterial species they encounter.

Several serogroups of *D. nodosus* on individual feet have been reported elsewhere (Claxton et al., 1983; Hindmarsh and Fraser, 1985; Jelinek et al., 2000; Moore et al., 2005) but not using the typing methods used in the current study where both *pgr* data and MLVA provided evidence for the occurrence of multiple strains in most animals studied. Not all the *D. nodosus* strains detected on the feet of lambs were on their mother's feet, indicating that the strains detected on lambs’ feet originated from sources additional to their mothers’ feet, most likely contaminated bedding in the communal pen. In addition, not all the strains detected in ewes were present in their offspring. It is possible that the specific variants on the ewes were not detected by chance, or were present below the minimum detection level or that only some of the strains were transferred via bedding.

Given the survival time of *D. nodosus* off host and the *D. nodosus*-negative stored straw samples, the most probable source of *D. nodosus* was the population of ewes in the communal pen. Methods to reduce the load of *D. nodosus* in ewes and the environment would probably have the biggest impact on transmission to newborn lambs. Management strategies that have been linked to a reduction in footrot prevalence and incidence include rapid appropriate treatment of diseased sheep (Kaler et al., 2010; Wassink et al., 2010), segregation of diseased sheep and footbathing healthy sheep (Wassink et al., 2004).

The identification of transmission routes and understanding the role of the environment is critical for the control of footrot. Previous studies have highlighted that diseased sheep are a reservoir of infection (Green et al., 2007; Kaler et al., 2010; Smith et al., 2014; Whittington, 1995), although transmission occurs indirectly via contaminated pasture or floors (Beveridge, 1941; Whittington, 1995). The current study has demonstrated that the environment potentially forms at least a temporary reservoir of infection for lambs because *D. nodosus* was detected in the straw bedding in the communal pens, and lambs have strains of *D. nodosus* on their feet not detected on their mother's feet.

The fact that lambs were *D. nodosus*-negative at birth suggests that it might be possible to produce *D. nodosus*-free lambs without the need for a caesarean birth. This finding is of interest to those performing challenge studies on pathogen-free individuals. At the practical, farm level this information might also be useful in countries that have eradicated footrot. In countries where footrot is endemic, even if lambs could be kept *D. nodosus*-free around lambing time, it is highly unlikely that this status could be maintained because of the high levels of infection in ewes. Reducing initial exposure (as suggested above) might be beneficial to subsequent disease severity, however, it might be detrimental if later age at first exposure increases disease severity.

## 5 Conclusions

We have provided evidence that lambs are born *D. nodosus*-negative, but within hours of birth several strains of *D. nodosus* are detectable on their feet. The strains detected were a combination of those present on their mother's feet and on the feet of other ewes. Straw bedding in the communal pen was *D. nodosus*-positive and the most likely source of *D. nodosus* for newborn lambs.

## Conflict of interest

None of the authors have any conflict of interest.

## Figures and Tables

**Table 1:** All primers and probes used in the study.

Primer (5′–3′)	Sequence	Expected size in VCS1703A (BP)	Reference
Cc	TCGGTACCGAGTATTTCTACCCAACACCT	783	La Fontaine et al. (1993)
Ac	CGGGGTTATGTAGCTTGC	783	La Fontaine et al. (1993)
27F	AGAGTTTGATCMTGGCTCAG	1500	Lane (1991); Baker et al. (2003)
1525R	AAGGAGGTGWTCCARCC	1500	Lane (1991); Baker et al. (2003)
*pgrAF1*	CCTGCACCATGCTTGTTAAA	290	Calvo-Bado et al. (2011a)
*pgrAR1*	GCTGTTGGTGGTTTGGCTAT	290	Calvo-Bado et al. (2011a)
M13F	GTAAAACGACGGCCAG	N/A	Supplied in the cloning kit
M13R	CAGGAAACAGCTATGAC	N/A	Supplied in the cloning kit
DNTR02F	(FAM)-GATCCATCGTTTCATCGTCA	549	Russell et al. (2014)
DNTR02R	CGCACTTTAGCCGTTATGTTT	549	Russell et al. (2014)
DNTR09F	(VIC)-GGCGTAAACGAAATGCCTAA	987	Russell et al. (2014)
DNTR09R	ATCGGCGGAAGATTGTCTC	987	Russell et al. (2014)
DNTR10F	(NED)-CCGTCTATCCACCCGATTTA	626	Russell et al. (2014)
DNTR10R	TTGAACCGCGTCACTATCAG	626	Russell et al. (2014)
DNTR19F	(PET)-CCCGTCGAATCACTCCAG	854	Russell et al. (2014)
DNTR19R	GGTAGCGCCGAAGAAAGA	854	Russell et al. (2014)
*rpoDF*	GCTCCCATTTCGCGCATAT	61	Calvo-Bado et al. (2011b)
*rpoDR*	CTGATGCAGAAGTCGGTAGAACA	61	Calvo-Bado et al. (2011b)
*rpoD* Taqman probe	(6FAM)-CATTCTTACCGGKCG-(BBQ)	61	Calvo-Bado et al. (2011b)
*pgrAF*	CATGAATGATAATATTTACCTTTTCGTT	298	
*pgrAR*	AAGATTGATGATGCTCCAGAAGAAG	298	
*pgrA* Taqman probe	(6FAM)-CCTGCACCATGCTTGTTAAACTCT AATTTT-(BBQ)	298	
*pgrBF*	AAAGGTGATCTCAACTGTATCGTCAT	N/A	
*pgrBR*	AATYARCARMGCCARAATTAGAGCTTAAT	N/A	
*pgrB* Taqman probe	(6FAM)-TTTACCCGCACCGTKCT-(BBQ)	N/A	

*FAM – Carboxyfluorescein*, BBQ (Black Berry Quencher). BP is the size of fragment in base pairs.

**Table 2:** Distribution of *pgrA* R1 tandem repeats in five pairs of ewes and lambs (14 ewe and 10 lamb feet).

Ewe/Lamb ID	Number of clones sequenced	Number of *pgrA* tandem repeats in the R1 region
**E 1**	6	3, 4, 5, 11, 13, 16
**L 1**	4	4, 11, 12, 16
**E 2**	3	4, 11, 15
**L 2**	5	3, 4, 6, 11, 16
**E 3**	4	6, 15, 16, 21
**L 3**	2	16, 20
**E 4**	4	4, 6, 11, 13
**L 4**	5	4, 6, 11, 12, 16
**E 5**	4	5, 12, 15, 16
**L 5**	5	4, 11, 12, 15, 16

**Table 3:** DNTR19 and DNTR10 allelic distribution between six and two pairs of ewes and lambs.

ID	DNTR19							DNTR10					
	3+	4	5	6	7	8	Total	3	4	7	9	10	Total
**E 1**	1	1	1	1	1	1	6	1	1	1	1	1	5
**L 1**	0	0	0	1	0	0	1	1	0	0	0	0	1
**E 2**	1	1	0	1	0	0	3	1	0	0	1	0	2
**L 2**	1	0	0	0	0	0	1	1	0	0	0	0	1
**E 3**	0	1	0	0	0	0	1						
**L 3**	1	0	0	0	0	0	1						
**E 4**	0	1	1	0	0	0	2						
**L 4**	0	0	0	1	0	0	1						
**E 5**	1	1	1	1	1	0	5						
**L 5**	0	0	0	0	1	0	1						
**E 6**	0	1	0	0	0	0	1						
**L 6**	0	0	0	0	0	1	1						

+ The numbers given in the table heading are the number of tandem repeats.
