# Supplementary material for: The role of the environment in transmission of Dichelobacter nodosus between ewes and their lambs
Source: Vet Microbiol. 2015 Aug 31;179(1-2):53–9. doi: 10.1016/j.vetmic.2015.04.010 (PMC4518504; doi:10.1016/j.vetmic.2015.04.010)
Supplement: Supplementary file 1 [file mmc1.pdf]

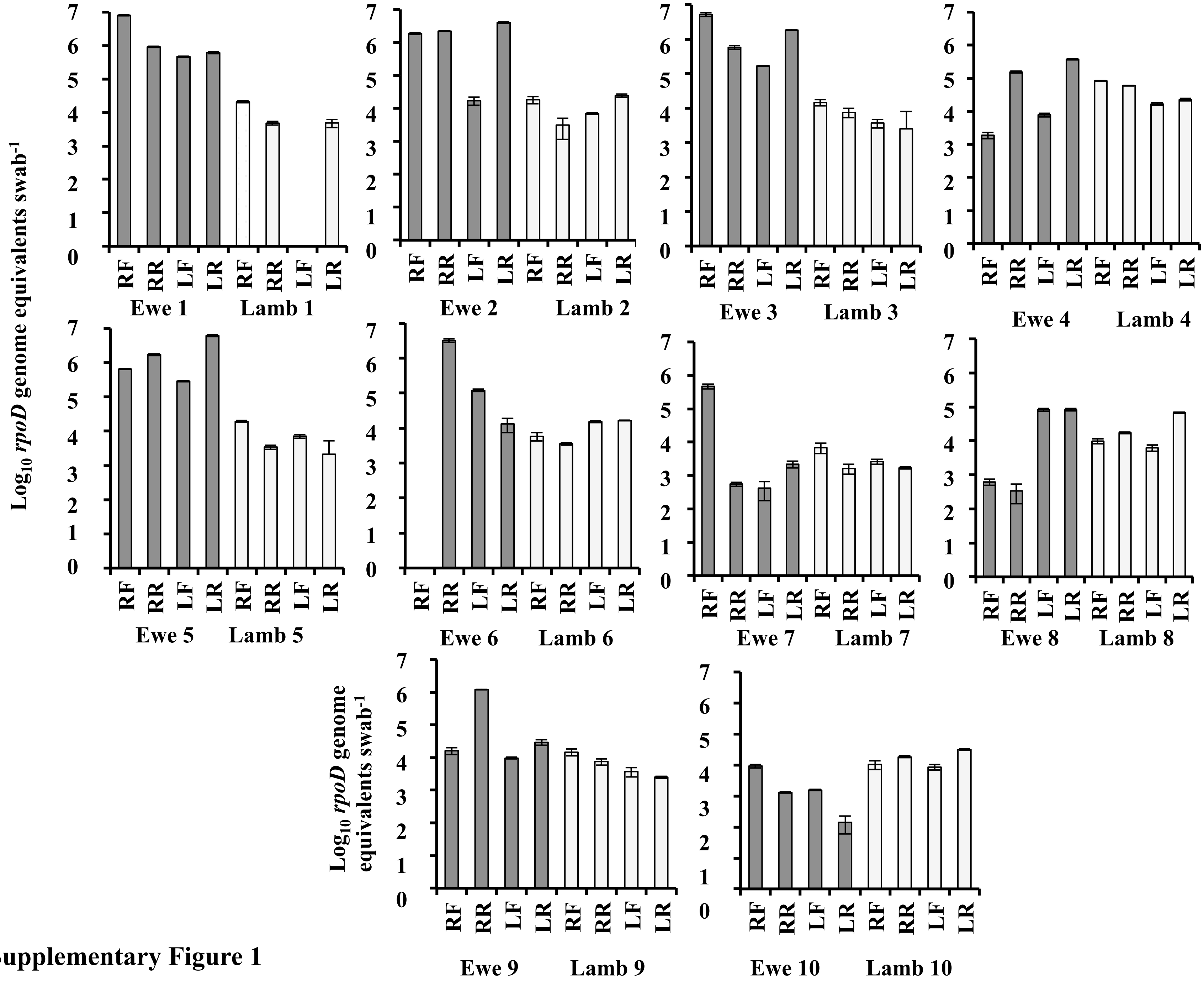

Supplementary Figure 1

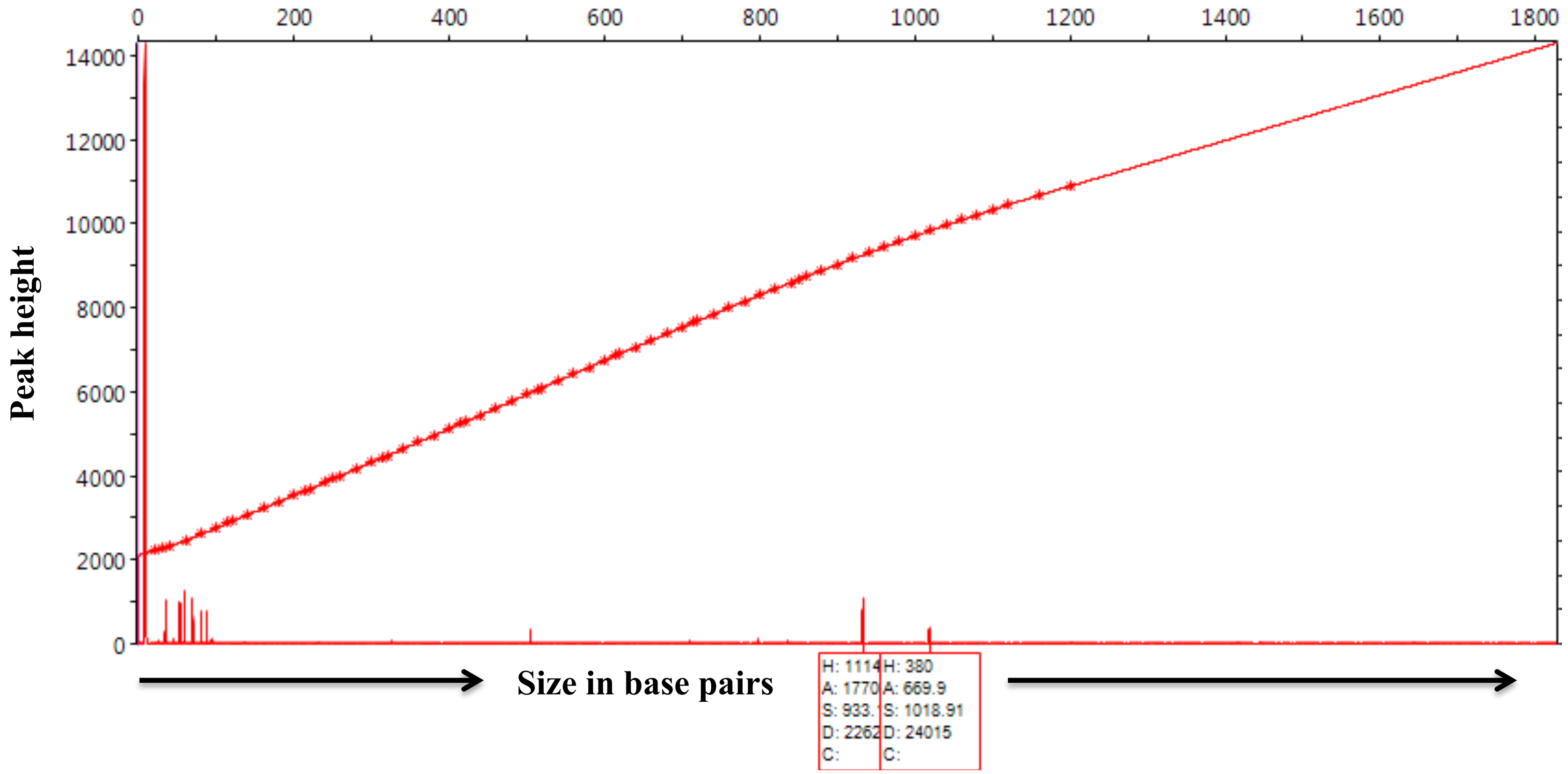

Presence of DNTR19 alleles in Ewe 1. DNTR19 alleles were present on the left front foot in Ewe 1. The size of the alleles is 933.18 and 1018.91 base pairs that correspond to 5 and 6 tandem repeats respectively. The additional peaks in the figure were not analysed due to their occurrence before the cut off size of 500 bp. The asterisk on the slope are different size fragments of GeneScan 1200 LIZ dye size standard.

Supplementary Figure 2
