# Supplementary material for: The role of the environment in transmission of Dichelobacter nodosus between ewes and their lambs
Source: Vet Microbiol. 2015 Aug 31;179(1-2):53–9. doi: 10.1016/j.vetmic.2015.04.010 (PMC4518504; doi:10.1016/j.vetmic.2015.04.010)
Supplement: Supplementary file 2 [file mmc2.docx]

**Table 1: All primers and probes used in the study**

| **Primer (5’-3’)** | **Sequence** | **Expected size in VCS1703A (BP)** | **Reference** |
| --- | --- | --- | --- |
| Cc | TCGGTACCGAGTATTTCTACCCAACACCT | 783 | ([La Fontaine et al., 1993](#_ENREF_15)) |
| Ac | CGGGGTTATGTAGCTTGC | 783 | ([La Fontaine et al., 1993](#_ENREF_15)) |
| 27F | AGAGTTTGATCMTGGCTCAG | 1500 | (Lane, 1991); (Baker et al., 2003) |
| 1525R | AAGGAGGTGWTCCARCC | 1500 | (Lane, 1991); (Baker et al., 2003) |
| *pgrAF1* | CCTGCACCATGCTTGTTAAA | 290 | (Calvo-Bado et al., 2011a) |
| *pgrAR1* | GCTGTTGGTGGTTTGGCTAT | 290 | (Calvo-Bado et al., 2011a) |
| M13F | GTAAAACGACGGCCAG | N/A | Supplied in the cloning kit |
| M13R | CAGGAAACAGCTATGAC | N/A | Supplied in the cloning kit |
| DNTR02F | (6FAM)-GATCCATCGTTTCATCGTCA | 549 | (Russell et al., 2014) |
| DNTR02R | CGCACTTTAGCCGTTATGTTT | 549 | (Russell et al., 2014) |
| DNTR09F | (VIC)-GGCGTAAACGAAATGCCTAA | 987 | (Russell et al., 2014) |
| DNTR09R | ATCGGCGGAAGATTGTCTC | 987 | (Russell et al., 2014) |
| DNTR10F | (NED)-CCGTCTATCCACCCGATTTA | 626 | (Russell et al., 2014) |
| DNTR10R | TTGAACCGCGTCACTATCAG | 626 | (Russell et al., 2014) |
| DNTR19F | (PET)-CCCGTCGAATCACTCCAG | 854 | (Russell et al., 2014) |
| DNTR19R | GGTAGCGCCGAAGAAAGA | 854 | (Russell et al., 2014) |
| *rpoDF* | GCTCCCATTTCGCGCATAT | 61 | (Calvo-Bado et al., 2011b) |
| *rpoDR* | CTGATGCAGAAGTCGGTAGAACA | 61 | (Calvo-Bado et al., 2011b) |
| *rpoD* Taqman probe | (6FAM)-CATTCTTACCGGKCG-(BBQ) | 61 | (Calvo-Bado et al., 2011b) |
| *pgrAF* | CATGAATGATAATATTTACCTTTTCGTT | 298 |  |
| *pgrAR* | AAGATTGATGATGCTCCAGAAGAAG | 298 |  |
| *pgrA* Taqman probe | (6FAM)-CCTGCACCATGCTTGTTAAACTCT  AATTTT-(BBQ) | 298 |  |
| *pgrBF* | AAAGGTGATCTCAACTGTATCGTCAT | N/A |  |
| *pgrBR* | AATYARCARMGCCARAATTAGAGCTTAAT | N/A |  |
| *pgrB* Taqman probe | (6FAM)-TTTACCCGCACCGTKCT-(BBQ) | N/A |  |

FAM – Carboxyfluorescein, BBQ (Black Berry Quencher). BP is the size of fragment in base pairs.

| **Ewe/Lamb ID** |  | **Number of clones**  **sequenced** | **Number of *pgrA* tandem repeats in the R1 region** |  |
| --- | --- | --- | --- | --- |
| **Ewe 1** |  | 6 | 3, 4, 5, 11, 13, 16 |  |
| **Lamb 1** |  | 4 | 4, 11, 12, 16 |  |
| **Ewe 2** |  | 3 | 4, 11, 15 |  |
| **Lamb 2** |  | 5 | 3, 4, 6, 11, 16 |  |
| **Ewe 3** |  | 4 | 6, 15, 16, 21 |  |
| **Lamb 3** |  | 2 | 16, 20 |  |
| **Ewe 4** |  | 4 | 4, 6, 11, 13 |  |
| **Lamb 4** |  | 5 | 4, 6, 11, 12, 16 |  |
| **Ewe 5** |  | 4 | 5, 12, 15, 16 |  |
| **Lamb 5** |  | 5 | 4, 11, 12, 15, 16 |  |

**Table 2: Distribution of *pgrA* R1 tandem repeats in five pairs of ewes and lambs (14 ewe**

**and 10 lamb feet)**

| **ID** | **DNTR19** | | | | | | | | **DNTR10** | | | | | |
| --- | --- | --- | --- | --- | --- | --- | --- | --- | --- | --- | --- | --- | --- | --- |
|  | **3^+^** | **4** | **5** | **6** | **7** | **8** | **Total** | **3** | | **4** | **7** | **9** | **10** | **Total** |
| **E 1** | 1 | 1 | 1 | 1 | 1 | 1 | 6 | 1 | | 1 | 1 | 1 | 1 | 5 |
| **L 1** | 0 | 0 | 0 | 1 | 0 | 0 | 1 | 1 | | 0 | 0 | 0 | 0 | 1 |
| **E 2** | 1 | 1 | 0 | 1 | 0 | 0 | 3 | 1 | | 0 | 0 | 1 | 0 | 2 |
| **L 2** | 1 | 0 | 0 | 0 | 0 | 0 | 1 | 1 | | 0 | 0 | 0 | 0 | 1 |
| **E 3** | 0 | 1 | 0 | 0 | 0 | 0 | 1 |  |  |  |  |  |  |  |
| **L 3** | 1 | 0 | 0 | 0 | 0 | 0 | 1 |  |  |  |  |  |  |  |
| **E 4** | 0 | 1 | 1 | 0 | 0 | 0 | 2 |  | |  |  |  |  |  |
| **L 4** | 0 | 0 | 0 | 1 | 0 | 0 | 1 |  | |  |  |  |  |  |
| **E 5** | 1 | 1 | 1 | 1 | 1 | 0 | 5 |  | |  |  |  |  |  |
| **L 5** | 0 | 0 | 0 | 0 | 1 | 0 | 1 |  | |  |  |  |  |  |
| **E 6** | 0 | 1 | 0 | 0 | 0 | 0 | 1 |  | |  |  |  |  |  |
| **L 6** | 0 | 0 | 0 | 0 | 0 | 1 | 1 |  | |  |  |  |  |  |

**Table 3: DNTR19 and DNTR10 allelic distribution between six and two pairs of ewes and lambs.**

+ (The numbers given in the table heading are the number of tandem repeats)
